# Injecting drug use is associated with a more rapid CD4 cell decline among treatment naïve HIV-positive patients in Indonesia

**DOI:** 10.7448/IAS.17.1.18844

**Published:** 2014-01-03

**Authors:** Hinta Meijerink, Rudi Wisaksana, Shelly Iskandar, Martin den Heijer, Andre J A M van der Ven, Bachti Alisjahbana, Reinout van Crevel

**Affiliations:** 1 Department of Internal Medicine, Radboud University Nijmegen Medical Centre, Nijmegen, the Netherlands; 2 Department of Internal Medicine, Medical Faculty, Universitas Padjadjaran and Hasan Sadikin Hospital, Bandung, Indonesia; 3 Department of Psychiatry Medical Faculty, Universitas Padjadjaran and Hasan Sadikin Hospital, Bandung, Indonesia; 4 Department of Internal Medicine, Vrije Universiteit Amsterdam, Amsterdam, the Netherlands

**Keywords:** injecting drug use (IDU), CD4-positive T-Lymphocytes, cohort studies, human immunodeficiency virus, Indonesia

## Abstract

**Background:**

It remains unclear whether the natural course of human immunodeficiency virus (HIV) differs in subjects infected through injecting drug use (IDU) and no data have been published from low- or middle-income countries. We addressed this question in an urban cohort in Indonesia, which is experiencing a rapidly growing HIV epidemic strongly driven by IDU.

**Methods:**

All antiretroviral treatment (ART) naïve HIV-positive patients who had at least two subsequent CD4 cell counts available before starting ART were included in this study. We examined the association between IDU and CD4 cell decline using a linear mixed model, with adjustment for possible confounders such as HIV viral load and hepatitis C antibodies.

**Results:**

Among 284 HIV-positive ART naïve patients, the majority were male (56%) with a history of IDU (79% among men). People with a history of IDU had a statistically significant faster decline in CD4 cells (p<0.001). Based on our data, patients with a history of IDU would have an average 33% decline in CD4 cells after one year without ART, compared with a 22% decline among non-users. At two years, the decline would average 66 and 40%, respectively. No other factor was significantly associated with CD4 cell decline.

**Conclusions:**

We show that a history of IDU is associated with a more rapid CD4 cell natural decline among HIV-positive individuals in Indonesia. These findings have implications for monitoring ART naïve patients with a history of IDU and for starting ART in this group.

## Introduction

Injecting drug use (IDU) is responsible for human immunodeficiency virus (HIV) infections in 10% of all cases worldwide, and 30% of cases outside Africa [1]. IDU is not only a risk factor for HIV transmission but it may also change the natural course of HIV infection, for instance because of co-infections and/or nutritional deficiencies. In addition, heroin and other opioids have immune-modulating effects, which might alter the progression of HIV infection and/or susceptibility to infections [2–7].

Epidemiological studies looking at the association between IDU and the natural course of HIV infection have shown contradictory results [3,8–13]. Most studies used clinical endpoints, such as mortality and AIDS-free survival, and were conducted in high-income countries, mostly among Caucasian subjects [8–12]. However, factors unrelated to HIV infection may significantly skew the relation between IDU and AIDS-free survival. One such factor is mortality not related to HIV, which is usually higher among individuals injecting drugs [14–16]. Actually, various studies have shown that overdose and suicide rather than opportunistic infections are the main cause of death in HIV-positive drug users [17]. Furthermore, differences in the occurrence of AIDS defining illnesses, for example, Kaposi sarcoma, may also affect the association between risk group and AIDS-free survival [9].

HIV reduces the number of CD4+ T-lymphocytes (CD4 cells), thereby compromising cellular immunity. As such, the number of circulating CD4 cells is the primary marker for immunodeficiency in HIV-positive patients. Therefore, we determined the association between IDU and the natural decline of CD4 cells in HIV-positive ART naïve patients. The study was performed in a patient cohort in Indonesia, which has a rapidly growing HIV epidemic, largely driven by IDU [18]. To our knowledge, this is the first study addressing this issue in a low- or middle-income country.

## Methods

### Setting and study population

This study was embedded in a five-year programme (2006–11) called “IMPACT”, aimed at improving prevention, control and treatment of HIV among injecting drug users in West Java, Indonesia [18]. IMPACT has helped establish patient care in three clinics in Bandung, the capital of West Java (40 million people): a teaching hospital, a methadone clinic, and a prison clinic. In these clinics, people with and without a history of IDU, who are at risk for HIV infection or who present with signs and symptoms suggesting HIV/AIDS are counselled and tested for HIV. All testing is voluntary and informed consent is obtained from all study participants. HIV-positive patients are characterized and followed prospectively in a cohort study, which has been approved by the Health Research Ethics Committee at the Faculty of Medicine of Padjadjaran University/Dr. Hasan Sadikin General Hospital in Bandung, Indonesia. Data on demographic factors, history of IDU, co-morbidity, self-reported tuberculosis treatment and history of antiretroviral treatment (ART) are collected through interview with standard questionnaires. Laboratory examinations include CD4 cell measurement at baseline and fixed time points afterwards. Patients are seen by a doctor every 3–6 months if not on ART, and more frequently when ART is initiated. At the time of this study, ART was indicated in Indonesia for patients presenting with WHO clinical stage IV or a CD4 cell count<200 cells/µl in accordance with WHO guidelines from 2006. Since 2004, ART can be accessed free of charge in Indonesia.

### Data analysis and statistics

In this study, we selected all adults (≥16 years) presenting with HIV infection between August 2007 and August 2011, who had not yet been exposed to ART. To determine the association between IDU and the natural decline of CD4 cells over time, we included all patients who had two or more CD4 cell counts measured at least one month apart, with all measurements done before the start of ART. We used a linear mixed model to describe the decline of CD4 cells over time and to determine the effect association with IDU. To meet all criteria, we transformed all CD4 cell counts to the log scale, which enabled us to interpret the results of the mixed models as a relative change in CD4 cells after anti-logarithmic transformation. The dependent variable in our final model was the log (CD4 cells) at a certain time point. The independent variables were a constant estimate, time since baseline in months, IDU, and a quadratic interaction term between IDU and months. In addition, the model used a random intercept and random slope based on time since baseline. The model with the best fit was chosen, based on the likelihood-ratio test and Akaike information criterion (AIC) (the model with lowest AIC has the best fit). In addition, we examined possible confounders in the final model, such as age, gender, candida infection, hepatitis C virus (HCV) antibodies and HIV viral load. Each variable was added to the final model to examine whether it was associated with the CD4 cell decline or whether it was a confounder for the association with IDU. These models were compared using AIC. HCV viral loads were tested with RT-PCR in a selection of HCV antibody-positive individuals to distinguish currently active and previous (cleared) HCV infection. All statistical analyses were done using STATA statistical software (version 12.0).

## Results

From a total of 1500 HIV-positive ART naïve individuals, 284 met the inclusion criteria; 145 out of 743 (19.5%) individuals with a history of IDU and 123 out of 608 (20.2%) without. The majority were male (56%) with a history of IDU (79% among men) (Table 1). More than half (53.8%) of all patients were infected with HCV, and this was 89.6% among those with a history of IDU. Patients with and without a history of IDU had similar first CD4 cell counts (*p=*0.945) and similar average time between subsequent measurements (197 and 218 days, respectively; *p=*0.173). The total follow-up time between the first and last CD4 cell count before initiating ART was 379 days, which was not statistically different between those with and without a history of IDU (*p*=0.614).

**Table 1:** Characteristics of all HIV-positive patients with at least two subsequent CD4 cell counts without ART (N=284)

Variable	Total population N=284 % (n/N)	Without a history of IDU N=123 % (n/N)	With a history of IDU N=145 % (n/N)
Male	56.0 (159/284)	26.0 (32/123)	83.4 (121/145)
Senior high school or university	85.5 (194/227)	76.7 (92/120)	96.9 (94/97)
History of IDU	54.1 (145/268)	n.a.	n.a.
Hepatitis C infection	53.8 (135/251)	9.7 (10/103)	89.6 (121/135)
Candida infection	10.7 (22/206)	5.4 (6/111)	18.2 (16/88)
	Median (IQR)	Median (IQR)	Median (IQR)
Age (years)	28 (26–31)	27 (31–35)	28 (26–31)
Haemoglobin (g/dl)	13.7 (12.3–15.1)	12.8 (11.8–13.9)	14.5 (13.5–15.6)
CD4 cell count (cells/µl)	336 (237–459)	336 (249–444)	337 (222–493)
HIV viral load (log (copies/ml))	3.47 (2.85–4.06)	3.46 (2.46–3.80)	3.64 (3.01–4.38)

Characteristics of the study population according to history of IDU. All variables were measured at baseline.

N, number of patients; IQR, interquartile range; IDU, injecting drug use.

Almost half of the patients (49.3%) had two serial CD4 cell counts, 20.4% had three CD4 cell counts, and 16.6% had four CD4 cell counts. The remainder (13.7%) had more than five CD4 cell counts, with a maximum of 13 CD4 cell counts. The number of serial CD4 cell counts was similar between people with and without a history of IDU (*p*=0.803). The median CD4 cell count at baseline differed slightly with the number of serial CD4 cell counts available (324 cells/µl for two, 306 cells/µl for three, 407 cells/µl for four and 380 cells/µl for five or more serial CD4 cell counts; *p*=0.008).

The natural progression of CD4 cells in HIV-positive ART naïve patients could best be described with a model including CD4 cell count at baseline, IDU and time between the CD4 cell counts (Table 2). The final model was significantly better than a model without IDU (likelihood-ratio test χ^2^ 47.3=*p*<0.001). In this final model, the influence of IDU on the decline in CD4 cells was taken into account as a quadratic factor, which fit significantly better than the model without this quadratic factor (likelihood-ratio test χ^2^=48.3, *p*<0.001). In addition, we added a random slope to allow differences between subjects within the model (likelihood-ratio test χ^2^=609.9, *p*<0.001).

**Table 2:** Estimated parameters of the final mixed model describing the course CD4 cell decline in ART naïve HIV-positive patients (n=268)

Variable	Estimate	(95% CI)	*p*
Constant	0.0964	(0.0306; 0.1623)	0.004
Baseline CD4 cell count*	0.9623	(0.9364; 0.9882)	<0.001
IDU	−0.0083	(−0.0287; 0.0121)	0.427
Months	−0.0093	(−0.0133; −0.0054)	<0.001
IDU*months^2^	−0.0004	(−0.0005; −0.0003)	<0.001
Random effects parameters			
sd (months)	0.0287		
sd (constant)	3.42 e^−13^		

* All CD4 cell counts were transformed to log scale.

Months are the number of months since first CD4 cell count. Months^2^ is a quadratic term of time in months, to account for non-linear effects of time on CD4 cell decline. IDU is taken into the model as baseline variable to determine effect on the intercept and as interaction with months to examine the effect of IDU on the slope. This model with IDU fit better than the model without IDU: likelihood-ratio test χ^2^=47.3; *p*<0.001.

ART, antiretroviral treatment; CI, confidence interval; IDU, injecting drug use; sd, standard deviation.

**Figure 1:**
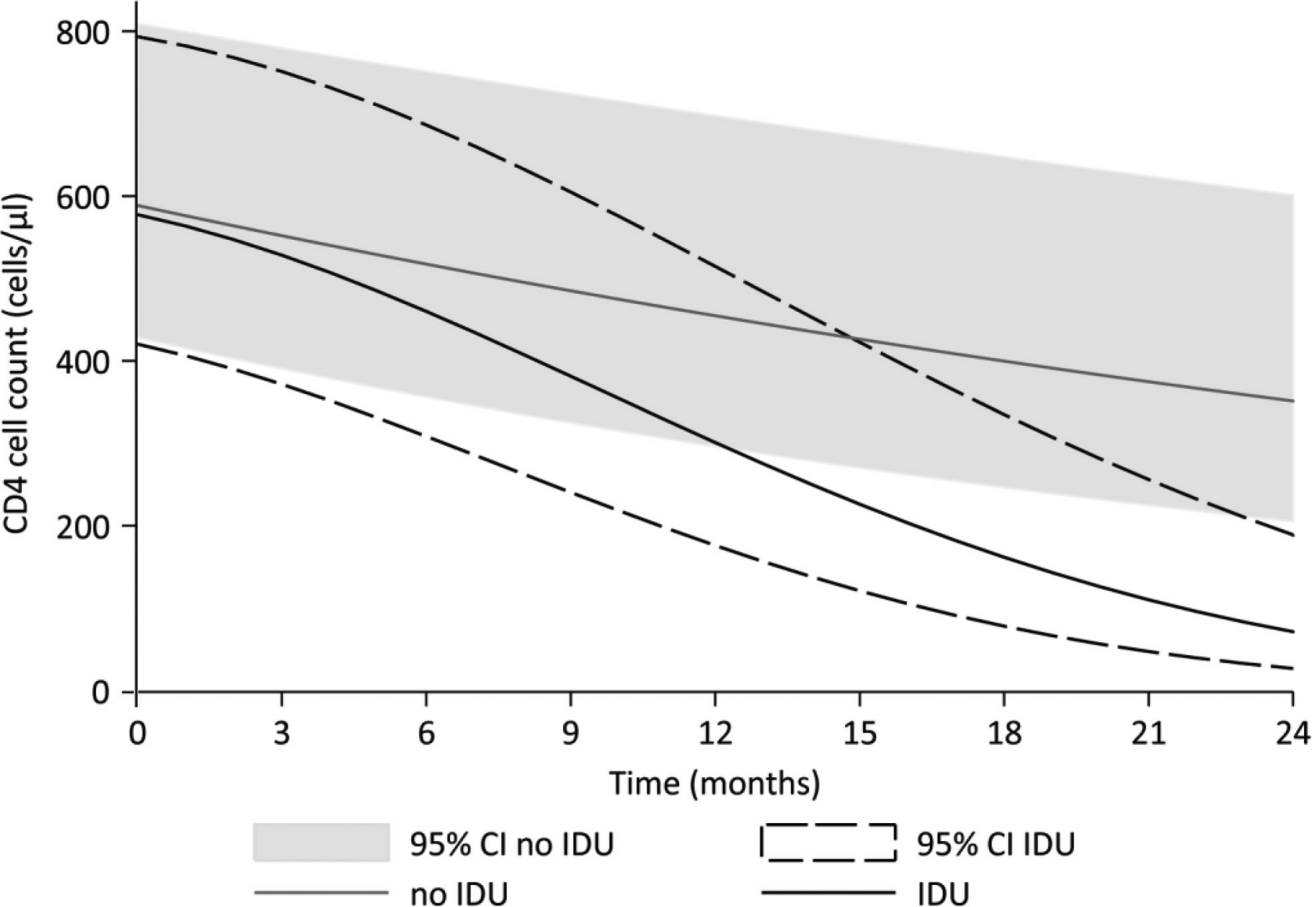
The predicted average decline in CD4 cells of HIV-positive ART naïve patients for people without (grey) and with (black) a history of injecting drug use (IDU). The 95% confidence intervals are given for people without (grey shaded area) and with (black dashed lines) a history of IDU. The CD4 cells were predicted with the following model: Log (CD4 cells)=0.0964+0.9623*log (CD4 cells baseline) – 0.0093*months – 0.0083*IDU – 0.0004 IDU*months^2^ (Table 2).

The estimates in Table 2 can be interpreted as relative change in CD4 cells with each unit increase in the variable; this means that the decline in CD4 cells can be noted as 1–10^estimate^. This translates in an average decline in CD4 cells of 2.1% per month among all HIV-positive ART naïve patients (1–10^−0.0093^). In addition, those with a history of IDU have an additional decline in CD4 cells of 0.09% decrease per month^2^. Based on our data, patients with a history of IDU would have an average 33% decline in CD4 cells after one year without ART, compared with a 22% decline among non-users. At two years, the decline would average 66 and 40%, respectively (Figure 1). Based on the model, the predicted CD4 cell decline after one year would be from 600 to 387 cells/µl for subjects with a history of IDU, and from 600 to 455 cells/µl for those without a history of IDU. After two years without ART, the predicted CD4 cell count in this model would be 196 cells/µl for those with a history of IDU, and 351 cells/µl for those without a history of IDU (Figure 1).

**Figure 2:**
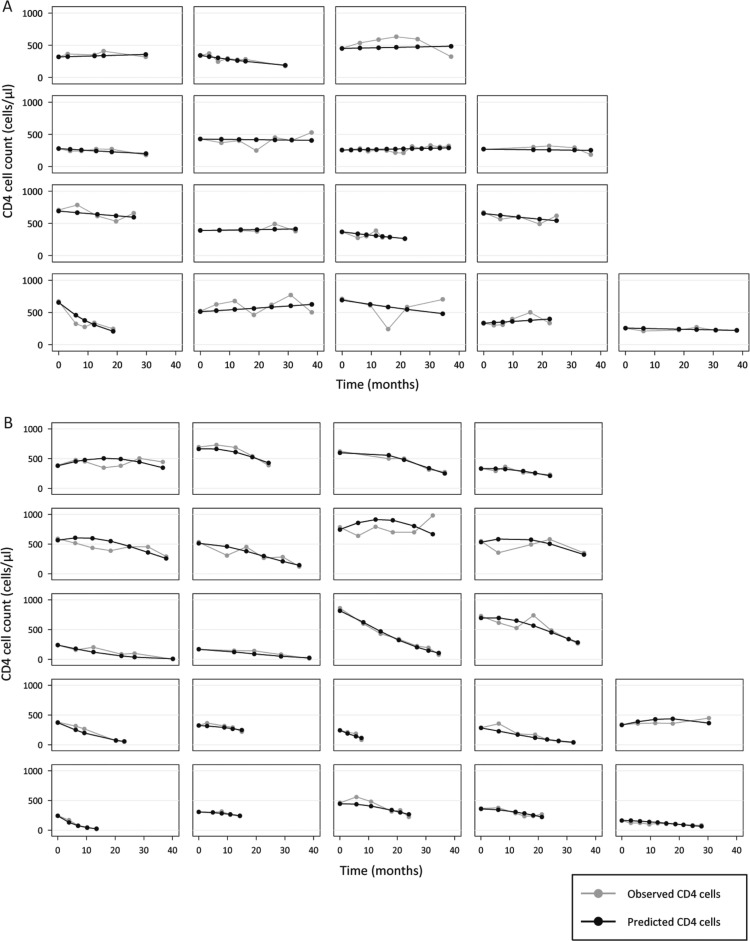
Natural CD4 cell decline of HIV-positive ART naïve patients, with observed (grey) and fitted (black) values for people without (A) and with (B) a history of injecting drug use (IDU). All subjects with at least five CD4 cell counts are shown.

Our model includes a random intercept and random slope for all individuals used to build this model. Figure 2 shows the fitted and observed values of all subjects with at least five CD4 cell measurements.

We also examined if this association could be explained by confounding. However, neither plasma HIV-RNA, gender, age, candidiasis and HCV infection could explain the association between IDU and CD4 cell decline (Table 3). Adding these variables, independently or combined, did not improve the overall fit of the model, as shown by the AIC, or alter the estimate of IDU (Table 3). HCV-status in our study was based on serological testing, which correlated relatively well with circulating HCV virus measured with PCR; among 70 individuals with HCV antibodies, 59 (84.4%) had a positive plasma HCV-RNA.

**Table 3:** The influence of other variables on the predicted CD4 cell decline

Variable	Estimate	*p*-value#	AIC	Estimate IDU*months^2^	*p*-Value IDU*months^2^
Complete model*	n.a.	n.a.	−677.8	−0.00043	<0.001
Plasma HIV-RNA	0.0001	0.977	−455.3	−0.00041	<0.001
Gender	−0.0014	0.915	−675.8	−0.00043	<0.001
Age	0.0011	0.274	−676.9	−0.00043	<0.001
Candida infection	0.0025	0.883	−640.7	−0.00043	<0.001
Hepatitis C antibodies	0.0055	0.764	−571.0	−0.00044	<0.001
All variables$	n.a.	n.a.	−385.0	−0.00045	<0.001

# *p*-value of the change in the complete model (as described in Table 2) by adding the variable.

* Model as described in Table 2: Log (CD4 cell count)=0.0964+0.9623*log (CD4 baseline) – 0.0093*months – 0.0083*IDU – 0.0004* IDU*months^2^.

$ Including all variables, namely plasma HIV-RNA, gender, age, Candida infection and hepatitis C antibodies, in the complete model.

Akaike information criterion (AIC) measures the relative goodness of fit of a statistical model; model with lowest AIC has the best fit.

n.a., not applicable.

## Discussion

In this study, we show that IDU is associated with a more rapid decline of CD4 cells among ART naïve HIV-positive patients in Indonesia. It should be noted that individuals with a history of IDU in our cohort are not from a lower or impoverished background that might affect disease progression; most had senior high school or university education. Instead, we suspect that the faster decline in CD4 cells among injecting drug users can be explained by biological factors, such as a direct effect of opioids, a difference in tropism or virulence of HIV virus transmitted via IDU, or underlying conditions such as hepatitis C virus. Irrespective of the exact mechanism, our findings indicate that HIV disease progression should be monitored carefully in injecting drug users.

We used CD4 cell decline as an accurate and meaningful parameter reflecting the natural course of HIV infection. Previous studies on the effect of IDU used clinical endpoints, which can be very different between low- and high-income countries [8–12]. In industrialized countries, violence and suicide are important causes of death among people who inject drugs, while in low-income countries tuberculosis is more prevalent [19]. In addition, the type of drug can differ: cocaine use among injecting drug users is rare in Indonesia [20] and common in high-income settings. Side effects of cocaine include stroke and myocardial infarction, which could also contribute to the clinical endpoints. For these reasons, CD4 cell decline is a more meaningful marker to investigate.

To our knowledge, this study is the first to show that IDU accelerates CD4 cell decline in a low-income setting. Our results support a study from McNeil *et al*. who used models of HIV progression with a square root transformation of CD4 cell counts in a high-income setting (Scotland) [12]. Similar to our cohort, 70% of patients in that study had a history of IDU and 70% were male. Based on their study population, the authors of that study created two models which both showed that individuals infected through IDU tended to have a faster CD4 cell decline than those infected through heterosexual contact (*p*=0.13 and 0.05) [12]. Other epidemiological studies on HIV progression in drug users found contradictory results [8,9,11,14,21]. For instance, men having sex with men had a higher AIDS incidence compared to other risk groups in a study using data from several cohorts. However, this effect could be explained by the high incidence of Kaposi sarcoma (often occurring at relatively higher CD4 cell counts) among men having sex with men [9]. Others found that the use of drugs, especially heroin, was associated with faster disease progression [11]. However, most studies were conducted in high-income settings, which have a different distribution of opportunistic infections, such as tuberculosis. In addition, many studies used clinical endpoints instead of CD4 cell counts, or other statistical methods, such as Markov models and hazard ratios. Consequently, the outcomes of these studies are somewhat difficult to compare with our results.

Older age has been associated with faster disease progression in several studies [9,22,23]. However, in our patient cohort the age range was small (IQR 26–31 years), which could explain why we did not find any association between age and the natural CD4 cell decline (*p*=0.312). HIV viral replication is another variable that may influence the natural decline of CD4 cells [8,24,25]. Plasma HIV RNA measurements, only introduced recently in Indonesia [26], were not associated with CD4 cell decline, and did not modify the association between IDU and CD4 cell decline.

HIV tropism has been associated with HIV progression, with faster CD4 cell decline and worse clinical outcome associated with the X4 virus [21,27,28]. X4 viruses have been found more often among recently infected injecting drug users, which could be explained by preferential infection of R5 virus through sexual intercourse [29,30]. However, another study found that X4 viruses were not more common among chronically infected drug users [29]. As we did not determine HIV tropism in our study, we cannot exclude a higher prevalence of X4 as a factor associated with faster CD4 cell decline.

Another possible explanation for the more rapid CD4 cell decline among IDUs could be the high rate of co-infection with HCV, as previous studies have shown deteriorated clinical outcome among HIV-positive individuals with HCV antibodies [31–36]. Indeed, the Swiss Cohort study found that both HCV seropositivity and IDU were independently associated with clinical outcome of HIV [34]. However, the relation between HCV infection and CD4 cell decline prior to ART remains unclear, [31,32,33,36,37] indeed the effects of HCV seems to be more pronounced among patients receiving ART [37,38]. In addition, no correlation has been reported between HCV and CD4 cell apoptosis, as a possible explanation for a more rapid CD4 cell decline [39]. In our study, the high prevalence (89.6%) of HCV infection among injecting drug users made it difficult to study the independent effect of HCV.

Finally, immunomodulating effects of heroin and other opioids could affect the CD4 cell decline. In vitro studies have indeed shown that opioids increase the expression of the HIV co-receptors CXCR4 and CCR5 and consequently increase viral replication of HIV [2–6]. In addition, opioids could influence HIV-mediated apoptosis of both HIV-infected and uninfected cells, which is considered an important cause of CD4 cell decline during HIV infection. In neuronal cells, morphine was shown to enhance HIV-induced apoptosis [40,41]. Also in immune cells (peripheral blood mononuclear cells), significantly more apoptosis was observed when cells were exposed to morphine in combination with HIV protein gp120 [42].

In this study, we only selected subjects with at least two CD4 cell counts before initiating ART. Only 20% of our cohort fits these criteria, since many patients come at a late stage of disease and start ART soon after diagnosis. However, there were no differences between individuals with and without a history of IDU in terms of proportion of subjects included, baseline characteristics and rates of loss to follow-up. Therefore, it seems unlikely that the difference we found in CD4 cell decline was due to selection bias. Also, patient characteristics were comparable with those of the total population, suggesting that our study population is representative of patients diagnosed with HIV in this setting. However, active drug users who are HIV positive (and who might have a different prognosis) probably have lower access to health services in Indonesia, and might therefore be underrepresented. Overall, we believe selection bias within the HIV clinic was limited, but we cannot exclude bias due to differences in health seeking behaviour.

## Conclusions

In conclusion, we have shown that IDU accelerates the natural decline of CD4 cells in HIV patients in Indonesia. In a recent study within the same cohort, we have found that a history of IDU in HIV-positive patients does not have a negative effect on virological or immunological response to ART, nor on retention to treatment and mortality [18]. These findings indicate that HIV disease progression should be monitored carefully in injecting drugs users and that earlier initiating of ART should be considered for this risk group.

## References

[1] UNAIDS (2007). Injecting drug use: focussed HIV prevention works. http://www.unaids.org/en/Resources/PressCentre/Featurestories/2007/May/20070511BPHighcoveragesites.

[2] Finley MJ, Happel CM, Kaminsky DE, Rogers TJ (2008). Opioid and nociceptin receptors regulate cytokine and cytokine receptor expression. Cell Immunol.

[3] Kapadia F, Vlahov D, Donahoe RM, Friedland G (2005). The role of substance abuse in HIV disease progression: reconciling differences from laboratory and epidemiologic investigations. Clin Infect Dis.

[4] Wang J, Barke RA, Ma J, Charboneau R, Roy S (2008). Opiate abuse, innate immunity, and bacterial infectious diseases. Arch Immunol Ther Exp.

[5] Donahoe RM, Vlahov D (1998). Opiates as potential cofactors in progression of HIV-1 infections to AIDS. J Neuroimmunol.

[6] Steele AD, Henderson EE, Rogers TJ (2003). Mu-opioid modulation of HIV-1 coreceptor expression and HIV-1 replication. Virology.

[7] Friedman H, Pross S, Klein TW (2006). Addictive drugs and their relationship with infectious diseases. FEMS Immunol Med Microbiol.

[8] Brettle RP, McNeil AJ, Burns S, Gore SM, Bird AG, Yap PL (1996). Progression of HIV: follow-up of Edinburgh injecting drug users with narrow seroconversion intervals in 1983–1985. AIDS.

[9] CASCADE (2000). Time from HIV-1 seroconversion to AIDS and death before widespread use of highly-active antiretroviral therapy: a collaborative re-analysis. Collaborative Group on AIDS Incubation and HIV Survival including the CASCADE EU Concerted Action. Concerted Action on SeroConversion to AIDS and Death in Europe. Lancet.

[10] Cozzi Lepri A, Pezzotti P, Dorrucci M, Phillips AN, Rezza G (1994). HIV disease progression in 854 women and men infected through injecting drug use and heterosexual sex and followed for up to nine years from seroconversion. Italian Seroconversion Study. BMJ.

[11] Ronald PJ, Robertson JR, Elton RA (1994). Continued drug use and other cofactors for progression to AIDS among injecting drug users. AIDS.

[12] McNeil AJ (1997). Bayes estimates for immunological progression rates in HIV disease. Stat Med.

[13] Rivera-Amill V, Silverstein PS, Noel RJ, Kumar S, Kumar A (2010). Morphine and rapid disease progression in nonhuman primate model of AIDS: inverse correlation between disease progression and virus evolution. J Neuroimmune Pharmacol.

[14] Hendriks JC, Satten GA, van Ameijden EJ, van Druten HA, Coutinho RA, van Griensven GJ (1998). The incubation period to AIDS in injecting drug users estimated from prevalent cohort data, accounting for death prior to an AIDS diagnosis. AIDS.

[15] Prins M, Veugelers PJ (1997). Comparison of progression and non-progression in injecting drug users and homosexual men with documented dates of HIV-1 seroconversion. European Seroconverter Study and the Tricontinental Seroconverter Study. AIDS.

[16] van Haastrecht HJ, van Ameijden EJ, van den Hoek JA, Mientjes GH, Bax JS, Coutinho RA (1996). Predictors of mortality in the Amsterdam cohort of human immunodeficiency virus (HIV)-positive and HIV-negative drug users. Am J Epidemiol.

[17] Hulse GK, English DR, Milne E, Holman CD (1999). The quantification of mortality resulting from the regular use of illicit opiates. Addiction.

[18] Wisaksana R, Indrati AK, Fibriani A, Rogayah E, Sudjana P, Djajakusumah TS (2010). Response to first-line antiretroviral treatment among human immunodeficiency virus-infected patients with and without a history of injecting drug use in Indonesia. Addiction.

[19] Degenhardt L, Hall W, Warner-Smith M (2006). Using cohort studies to estimate mortality among injecting drug users that is not attributable to AIDS. Sex Transm Infect.

[20] Iskandar S, Basar D, Hidayat T, Siregar IM, Pinxten L, van Crevel R (2010). High risk behavior for HIV transmission among former injecting drug users: a survey from Indonesia. BMC Public Health.

[21] Nozza S, Canducci F, Galli L, Cozzi-Lepri A, Capobianchi MR, Ceresola ER (2012). Viral tropism by geno2pheno as a tool for predicting CD4 decrease in HIV-1-infected naive patients with high CD4 counts. J Antimicrob Chemother.

[22] Darby SC, Ewart DW, Giangrande PL, Spooner RJ, Rizza CR (1996). Importance of age at infection with HIV-1 for survival and development of AIDS in UK haemophilia population. UK Haemophilia Centre Directors’ Organisation. Lancet.

[23] UK Register of HIV Seroconverters Steering Committee (1998). The AIDS incubation period in the UK estimated from a national register of HIV seroconverters. UK Register of HIV Seroconverters Steering Committee. AIDS.

[24] Mellors JW, Munoz A, Giorgi JV, Margolick JB, Tassoni CJ, Gupta P (1997). Plasma viral load and CD4+ lymphocytes as prognostic markers of HIV-1 infection. Ann Intern Med.

[25] Rodriguez B, Sethi AK, Cheruvu VK, Mackay W, Bosch RJ, Kitahata M (2006). Predictive value of plasma HIV RNA level on rate of CD4 T-cell decline in untreated HIV infection. JAMA.

[26] Fibriani A, Farah N, Kusumadewi I, Pas SD, van Crevel R, van der Ven A (2012). Low cost HIV-1 quantitative RT-PCR assay in resource-limited settings: improvement and implementation. J Virol Methods.

[27] Waters L, Mandalia S, Randell P, Wildfire A, Gazzard B, Moyle G (2008). The impact of HIV tropism on decreases in CD4 cell count, clinical progression, and subsequent response to a first antiretroviral therapy regimen. Clin Infect Dis.

[28] Goetz MB, Leduc R, Kostman JR, Labriola AM, Lie Y, Weidler J (2009). Relationship between HIV coreceptor tropism and disease progression in persons with untreated chronic HIV infection. J Acquir Immune Defic Syndr.

[29] Monno L, Scudeller L, Ladisa N, Maggi P, Angarano G (2011). A greater prevalence of X4 viruses in HIV type 1 intravenous drug users reflects a “CD4+ effect”. AIDS research and human retroviruses.

[30] de Mendoza C, Rodriguez C, Garcia F, Eiros JM, Ruiz L, Caballero E (2007). Prevalence of X4 tropic viruses in patients recently infected with HIV-1 and lack of association with transmission of drug resistance. J Antimicrob Chemother.

[31] Piroth L, Duong M, Quantin C, Abrahamowicz M, Michardiere R, Aho LS (1998). Does hepatitis C virus co-infection accelerate clinical and immunological evolution of HIV-infected patients?. AIDS.

[32] Piroth L, Grappin M, Cuzin L, Mouton Y, Bouchard O, Raffi F (2000). Hepatitis C virus co-infection is a negative prognostic factor for clinical evolution in human immunodeficiency virus-positive patients. J Viral Hepat.

[33] Carlos Martin J, Castilla J, Lopez M, Arranz R, Gonzalez-Lahoz J, Soriano V (2004). Impact of chronic hepatitis C on HIV-1 disease progression. HIV Clin Trials.

[34] Greub G, Ledergerber B, Battegay M, Grob P, Perrin L, Furrer H (2000). Clinical progression, survival, and immune recovery during antiretroviral therapy in patients with HIV-1 and hepatitis C virus coinfection: the Swiss HIV Cohort Study. Lancet.

[35] De Luca A, Bugarini R, Lepri AC, Puoti M, Girardi E, Antinori A (2002). Coinfection with hepatitis viruses and outcome of initial antiretroviral regimens in previously naive HIV-infected subjects. Arch Intern Med.

[36] Stebbing J, Waters L, Mandalia S, Bower M, Nelson M, Gazzard B (2005). Hepatitis C virus infection in HIV type 1-infected individuals does not accelerate a decrease in the CD4+ cell count but does increase the likelihood of AIDS-defining events. Clin Infect Dis.

[37] Potter M, Odueyungbo A, Yang H, Saeed S, Klein MB (2010). Impact of hepatitis C viral replication on CD4+ T-lymphocyte progression in HIV-HCV coinfection before and after antiretroviral therapy. AIDS.

[38] Chen TY, Ding EL, Seage Iii GR, Kim AY (2009). Meta-analysis: increased mortality associated with hepatitis C in HIV-infected persons is unrelated to HIV disease progression. Clin Infect Dis.

[39] Korner C, Kramer B, Schulte D, Coenen M, Mauss S, Fatkenheuer G (2009). Effects of HCV co-infection on apoptosis of CD4+ T-cells in HIV-positive patients. Clin Sci.

[40] Hu S, Sheng WS, Lokensgard JR, Peterson PK (2005). Morphine potentiates HIV-1 gp120-induced neuronal apoptosis. J Infect Dis.

[41] Gurwell JA, Nath A, Sun Q, Zhang J, Martin KM, Chen Y (2001). Synergistic neurotoxicity of opioids and human immunodeficiency virus-1 Tat protein in striatal neurons in vitro. Neuroscience.

[42] Moorman J, Zhang Y, Liu B, LeSage G, Chen Y, Stuart C (2009). HIV-1 gp120 primes lymphocytes for opioid-induced, beta-arrestin 2-dependent apoptosis. Biochim Biophys Acta.

